# Inconclusive Evidence in Support of the Dopamine Hypothesis of Psychosis: Why Neurobiological Research Must Consider Medication Use, Adjust for Important Confounders, Choose Stringent Comparators, and Use Larger Samples

**DOI:** 10.3389/fpsyt.2018.00174

**Published:** 2018-05-01

**Authors:** Michael P. Hengartner, Joanna Moncrieff

**Affiliations:** ^1^Department of Applied Psychology, Zurich University of Applied Sciences, Zurich, Switzerland; ^2^Division of Psychiatry, University College London, London, United Kingdom

**Keywords:** dopamine hypothesis, schizophrenia, psychosis, bias, confounding, substance abuse, stress

Despite several inconsistencies and methodological biases (1), the dopamine hypothesis (DH) remains a popular topic in schizophrenia research. In its current version III, the DH asserts that environmental stress and substance abuse, in interaction with a genetic susceptibility, lead to dopamine dysregulation, and that increases in striatal presynaptic dopamine concentration causes psychosis (or proneness to psychosis) through a process of aberrant salience to external stimuli (2). Recently, Jauhar et al. (3) examined the putative role of striatal dopamine synthesis capacity in patients with bipolar disorders with current or previous psychotic episode compared to patients with first-episode schizophrenia and healthy controls. Though this study and similar others (for a review, see 2) may show an association between the dopaminergic system and psychosis, these findings cannot provide convincing evidence in support of the DH due to several methodological limitations. In the following we will outline these biases by using Jauhar et al. (3) as a benchmark study. However, the same issues likewise apply to other highly cited original research on the DH (e.g., 4, 5).

## Impact of preceding antipsychotic medication

Jauhar et al. (3) included people who have taken antipsychotics at some point prior to the scan, and some who were taking them at the time of the scan. Only just over half of the patients with psychotic disorders were antipsychotic naïve (10 of 22 in the bipolar group and 11 of 16 in the schizophrenia group), and results for this group are not presented separately. In another influential study on the DH conducted by Howes et al. (4), only 3 of 7 patients (43%) with schizophrenia were naïve to antipsychotic drugs before imaging, and in a study by McGowan et al. (5), all 16 patients with schizophrenia included in the study were acutely medicated with antipsychotics. This is problematic, because antipsychotics have a profound impact on dopaminergic pathways. That is, antipsychotics can cause progressive brain change (6), and neurobiological alterations have been demonstrated in animals (7) and healthy volunteers (8). It is likely that there are “carry-over” effects, such that patients who are described as “drug-free” but who have previous exposure cannot be assumed to have unaltered dopaminergic functioning (1). In support of this notion it has consistently been shown that use of psychotropic drugs in general (9) and antipsychotic drugs in particular (10) may persistently alter neurobiological functioning. Therefore, the effects of current or previous antipsychotic treatment, and other psychotropic drugs, cannot be easily ignored. Neurobiological characteristics attributed to psychosis may be drug-induced. A longitudinal study by Howes et al. (11) on striatal dopamine synthesis capacity in persons at-risk of psychosis is one of the few that enrolled antipsychotic naïve participants only and which found a prospective association between dopamine function and the subsequent onset of schizophrenia. However, as we will detail below, even in research with antipsychotic naïve participants there are several other limitations that question the validity of the reported findings.

## Confounding by environmental stress and substance abuse

Psychotic disorders are significantly influenced by environmental adversity, i.e., both acute and enduring stress, which in turn may impact on neurobiology (12). For instance, there is evidence that early-life poor parental care and acute psychological stress alter mesolimbic dopamine release in healthy volunteers (13). Substance abuse is another confounder, because it is frequent in psychotic patients, relates to environmental adversity, and interferes with the dopaminergic system (14). For instance, childhood trauma may increase ventral striatal dopamine responses to amphetamine use (15). The attentive reader will notice that we cite the same work as evidence against the DH that Howes and Kapur (2) refer to as supporting the DH. This is so because according to the DH version III, environmental stress and substance abuse increase striatal dopamine concentration, which is assumed to cause psychosis. We, likewise, acknowledge that substance abuse and stress impacts, among others, on dopaminergic pathways, but in contrast to Howes and Kapur (2) we disagree that striatal dopamine levels *cause* psychosis (or proneness to psychosis). Howes and Kapur (2) assume that dopamine dysfunction is part of the causal pathway leading to psychosis, but it is equally possible that the relationship between substance abuse/stress and dopamine as well as between substance abuse/stress and psychosis are independent processes, with the former relationship confounding analysis of an association between dopamine function and psychosis. Amphetamines, for instance, not only affect dopamine, but catecholamine in general and also serotonergic pathways (16), and the neurobiology of stress involves many more mechanisms than simply dopaminergic neurotransmission (17). Neuroinflammation and endocannabinoid signaling may be important substrates of the association between social stress and psychosis (18). In consequence, unless we account for the various neurobiological effects of substance abuse and environmental stress, we cannot know whether striatal dopamine concentration is directly and causally involved in psychosis or merely a spurious correlate.

## Power failure and sampling error

The number of participants with psychotic disorders and healthy controls in research on the DH is very small. The samples in Jauhar et al. (3), which are among the largest to date, comprised 22 patients with bipolar disorder, 16 patients with schizophrenia, and 22 healthy controls. In comparison, Howes et al. (4) included 24 patients with prodromal symptoms, 7 patients with schizophrenia, and 12 healthy controls, McGowan et al. (5) enrolled 16 patients with schizophrenia and 12 healthy controls, and Howes et al. (11) included 29 healthy controls, 9 at-risk persons who developed psychosis, and 15 at-risk persons who did not develop psychosis. These very small group sizes are a serious issue, because power failure does not only yield false-negative results, but, more importantly, it also produces inflated effect sizes and false-positive associations (19). Assume, for instance, that you want to determine the mean difference in IQ scores between men and women in a given population. All else being equal, study 1 enrols 10 men and 10 women, whereas study 2 samples 100 each. Even without proof it should be evident that, due to sampling error, the sex difference estimated in the smaller study 1, compared to study 2, is less accurate and more likely to be an over- or underestimation of the true difference, if there is any at all (for more details, see (19)). Because the underestimated and statistically insignificant group difference is unlikely to get published, it is the overestimated and statistically significant effect that enters the scientific literature. This form of selective reporting also explains why there are too many underpowered studies with statistically significant results in the psychiatric literature on brain volume abnormalities (20). These biases are rarely, if ever, appreciated in neurobiological research, but severely undermine the validity of neuroimaging studies on the DH.

## Inadequate comparators

There is potential bias associated with comparing extreme groups such as healthy controls to inpatients with schizophrenia (21, 22). Healthy controls are hardly comparable to people who have been admitted to hospital with acute psychosis. There are likely to be differences with respect to childhood adversity, socio-economic status, lifestyle (i.e., diet, exercise, substance abuse) and general physical health, but research on the DH typically matches controls to inpatients based on sex, age, and ethnicity only. A more stringent comparison would be to contrast patients with schizophrenia with patients who are equally distressed but non-psychotic, such as for instance acutely admitted patients with panic disorder or cluster C personality disorders. In contrast to healthy controls, patients with acutely distressing non-psychotic mental disorders are likely to be more comparable in terms of personal history of adversity, psychosocial impairments, and current levels of acute arousal and stress.

## Disconfirming evidence

There are two lines of evidence that challenge the DH. First, according to a comprehensive meta-analysis of randomized trials, antipsychotic medication does not prevent the development of schizophrenia in persons at ultra-high risk of psychosis (23). If increased striatal dopamine concentration were a necessary cause, then antipsychotic drugs should prevent the first onset of manifest psychosis in at-risk persons. Second, Howes and Kapur (2) state that if a psychopharmacological agent were to be found, that does not act upon the dopaminergic system and which effectively treats psychotic symptoms, then the DH would be rejected immediately. Although we are not aware of a drug that does not influence dopamine function at all, the effect of clozapine on D_2_ receptors is small compared to the effects of other antipsychotic agents. Specifically, its D_2_ binding affinity (expressed via the inhibition dissociation constant *K*_i_) is approximately 75 times less than risperidone and 100 times less than haloperidol (24). An analysis of the correlation between symptom reduction and the D_2_ dopamine blocking properties of different antipsychotics revealed clozapine to be an outlier (25). Yet clozapine is regarded as being as effective (26) if not more effective (27) than other antipsychotic agents. Therefore, it appears that clozapine's mechanism of action is largely independent of its effects on the dopamine system, and probably derives from its widespread effects on other neurotransmitter systems. Although these findings do not definitely disconfirm the DH, they suggest that dopamine function is neither a sufficient nor a necessary cause of psychosis.

## Conclusions

The commonly conducted experimental tests of the DH, such as the F-dopa uptake studies, are inconclusive due to several limitations. Larger sample sizes with antipsychotic naïve participants, adequate control of possible confounders and stringent comparators are necessary to provide a convincing test of the DH. If possible, researchers should measure striatal dopamine concentration before and after the onset of psychosis and statistically control for important covariates such as a person's activity, diet, and substance use, which impact on various neurobiological systems besides dopamine. Future work should also examine how stress, with its complex and multi-factorial brain mechanisms, may account for both striatal dopamine concentration and the experience of psychotic symptoms. Finally, the limitations outlined in this opinion paper also apply to other targets of drug action. Simply shifting the focus to another isolated neurotransmitter, such as for instance histamine, and analyzing its action in complete disregard of interactions between neurochemicals, complex neurological signaling networks and functional feedback loops, will not sufficiently advance our knowledge of the multiple, inter-related neurobiological pathways underlying psychosis.

## Author contributions

All authors listed, have made substantial, direct and intellectual contribution to the work, and approved it for publication.

### Conflict of interest statement

JM is a member and co-chair of the Critical Psychiatry Network. The other author declares that the research was conducted in the absence of any commercial or financial relationships that could be construed as a potential conflict of interest.
